# Supplementary figures and images for: Dual transcriptional activities of PAX3 and PAX7 spatially encode spinal cell fates through distinct gene networks
Source: PLoS Biol. 2025 Oct 24;23(10):e3003448. doi: 10.1371/journal.pbio.3003448 (PMC12574859; doi:10.1371/journal.pbio.3003448)

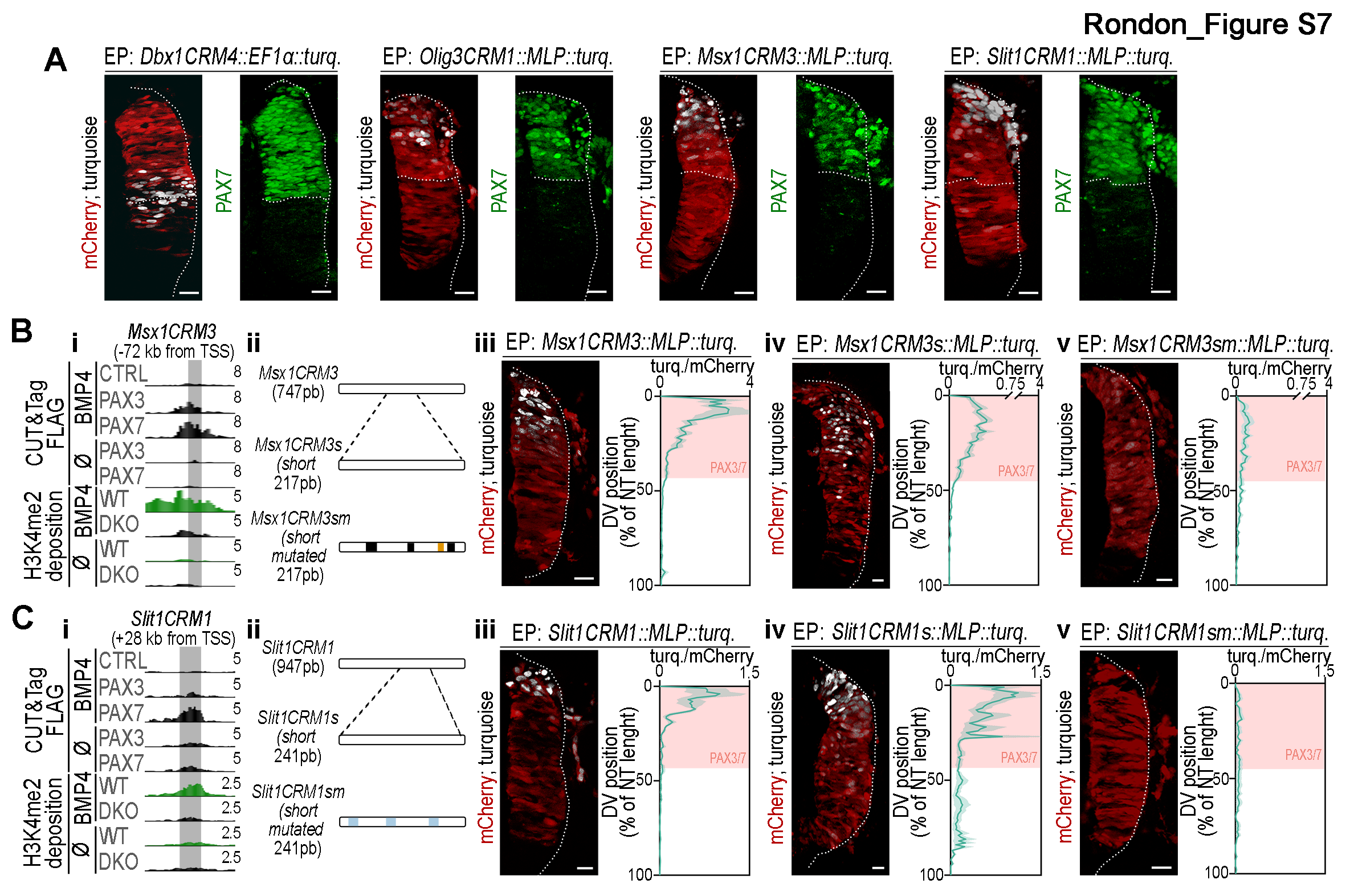

Supplement: S7 Fig — (A) Fluorescence of mCherry (red) and Turquoise (white), and immunodetection of PAX7 (green) on transverse sections of the chick neural tube, 24 h post-electroporation with the indicated constructs. Scale bars: 50 µm. (B, C) Panel i: UCSC Genome Browser screenshots showing normalised FLAG CUT&Tag read distributions (black tracks) in control (CTRL), FLAG-PAX3 (PAX3), FLAG-PAX7 (PAX7) day 5 organoids and H3K4me2 CUT&Tag read distributions (green tracks) in day6 wild-type (WT) and Pax3; Pax7 double-knockout (DKO) organoids, treated or untreated with BMP4. Read scales are shown in CPM. Grey bands highlight the genomic position of Msx1CRM3 (chr5:37895330-37896785) and Slit1CRM1 (chr19:41716058-41716783). Panel ii: Position of the shortened (s), or shortened and mutated (sm) versions of the Msx1CRM3 and Slit1CRM1. Mutated PAX motifs are highlighted in the CRM versions, with the following color scheme: orange for HD-HD, black for PrD, and light blue for PrD-HD. Panels iii–v: Fluorescence of mCherry (Red) and Turquoise (white) on transverse sections of the chick neural tube, 24 h post-electroporation with the pCAG-Cherry and the indicated constructs. Scale bars: 50 µm. Quantification of the ratio between Turquoise and mCherry signal intensities along the DV axis of the neural tube (NT), expressed as a percentage of NT length (bar plots represent mean ± s.e.m.; n > 4 embryos). Salmon-colored rectangles indicate the positions of PAX3/7-expressing progenitors. (TIF) [file pbio.3003448.s007.tif]
