# Supplementary material for: Dual transcriptional activities of PAX3 and PAX7 spatially encode spinal cell fates through distinct gene networks
Source: PLoS Biol. 2025 Oct 24;23(10):e3003448. doi: 10.1371/journal.pbio.3003448 (PMC12574859; doi:10.1371/journal.pbio.3003448)
Supplement: S3 Table — First square (Olig3CRM): Genomic position of Olig3CRM1; sequence of the shortened version of Olig3CRM1 (Olig3CRM1s), and mutated version of Olig3CRM1s. Second square (Msx1CRM): Genomic position of Msx1CRM3; sequence of the shortened version of Msx1CRM3 (Msx1CRM3s), and mutated version of Msx1CRM3s. Third square (Slit1CRM): Genomic position of Slit1CRM1; sequence of the shortened version of Slit1CRM1 (Slit1CRM1s), and mutated version of Slit1CRM1s. Fourth square (Dbx1CRM): Genomic position of Dbx1CRM4; sequence of the shortened version of Dbx1CRM4 (Dbx1CRM4s), and mutated version of Dbx1CRM4s. All squares: the PAX binding sites are highlighted in grey. (DOCX) [file pbio.3003448.s010.docx]

## Supplementary Table S3: Position and sequence of PAX motifs in PAX bound enhancers electroporated in chick embryos. Position and sequence of PAX motifs in PAX bound enhancers electroporated in chick embryos***.***

## First square (Olig3CRM): Genomic position of Olig3CRM1; sequence of the shortened version of Olig3CRM1 (Olig3CRM1s), and mutated version of Olig3CRM1s. Second square (Msx1CRM): Genomic position of Msx1CRM3; sequence of the shortened version of Msx1CRM3 (Msx1CRM3s), and mutated version of Msx1CRM3s. Third square (Slit1CRM): Genomic position of Slit1CRM1; sequence of the shortened version of Slit1CRM1 (Slit1CRM1s), and mutated version of Slit1CRM1s. Fourth square (Dbx1CRM): Genomic position of Dbx1CRM4; sequence of the shortened version of Dbx1CRM4 (Dbx1CRM4s), and mutated version of Dbx1CRM4s. All squares: the PAX binding sites are highlighted in grey.

***Olig3CRM1:*** chr10:19413882-19415316

***Olig3CRM1s:***TGTCTCCACTAACCCTAAACCTGTAGTGTCTTTGAAACCCTGCTGCAGGCTACCATTCTTAAGTTAAAGGAGTTTTGCTTTCGAAGAGGTACAAAGGAAACATCAAAGAAAGTGTCCTGGTAGTGACATAAGGACAGAATAATGGTGACCTAGCAGCTAGAGTCTTGGCCAAATGCTGAAAGTAATTCACGCCACTCACTAACAATGGGGCATGTGTGACAATGATATATACCAG

***Olig3CRM1sm::***TGTCTCCACTAACCCTAAACCTGTAGTGTCTTTGAAACCCTGCTGCAGGCTACCATTCTTAAGTTAAAGGAGTTTTGCTTTCGAAGAGGTACAAAGGAAACATCA**gg**GAAA**acact**CTGGTAGTGACATAAGGACAGAATAATGGTGACCTAGCAGCTAGAGTCTTGGCCAAATGCTGAAAGTAAT**ctgta**CCACTCA**tc**AAC**gg**TGGGGCATG**cacag**CAATGATATATACCAG

***Msx1CRM3 :*** chr5:37895412-37896158

***Msx1CRM3s:***CCTGTGCAAACTAAATTGCTGTGGACTGCTTAATTCAAAAGTGTTCCCTCGCGGGTGACAGCCCAGCAGTGACAAAATACAATTGGCAACAGGAGCCGGCAAGATCAAAGGCTGCCTATTGTAATGGCTTTGTTGCGTGAAGAATGGTGCA

***Msx1CRM3sm: :*** CCTGTGCAAACTAAATTGCT**tcc**GACTGCTTAATTCAAAAGTGTTCCCTCGCGGGTGACAGCCCAGCA**tcc**ACAAAATACAATTGGCAACAGGAGCCGGCAAGATCAAAGGCTGCCTATTG**gtca**GGCTTTGTTGC**tcc**AAGAATGGTGCA

***Slit1CRM1 :*** chr19:41715896-41716842

***Slit1CRM1s:*** CCAGCTATTTGTTTCAGTGGCGTAGCCTCCTGCCTCTGATGGGGGCTGGGCCCTGGCGGGACGGATGCTCCGTTCTTCTGACCAGTGCTGACAGTTTAATTAAACCCAGCTTGAGACGGGGTCATGGGGGTCAGTCATTCTTCCACAGGGCTGGGCAGGCTCCGCAGCCTGCCTCCCACCACCAGGGCCTTGTT

***Slit1CRM1sm:***

CCAGCTA**cc**TGTTTCAGTGG**tacga**CCTCCTGCCTCTGATGGGGGCTGGGCCCTGGCG**aggta**GATGCT**cc**GTTCTTCTGACCAGTGCTGACAGTTTAATTAAACCCAGCTTGAGACGGG**actgc**GGGGGTCAGTCA**cc**CTTCCACAGGGCTGGGCAGGCTCCGCAGCCTGCCTCCCACCACCAGGGCCTTGTT

##

***Dbx1CRM4 :*** chr7:49697106-49701929

***Dbx1CRM4s:*** CCAGCGCCACCTCACAGATGGAGGACCCAGTCTGTGGGAAAGAAGAGACCGTGACTTATCGGAACATATGGTTTCCTAAT

TCAAGTTAAAATGTCTGCACCCTGTTGCATCTGGTGCCATTTTAGTTAGTTAGAAATGCCACACAAGGCTGTGACTAAAG

GGGAAGAAACGGAGAAAAATACAGTTATTACACACTCAAGAGCTGGGTTTTGCGGCTCATTAGCCAGTGGCCTCCGTGAC

TAATTAGGTTTTGTCACTGTTGCGGGGTCATTATGCCTTAATTTTTTTGCATAGTTATTCAGCCGTCACTTGAGGA

***Dbx1CRM4sm:***

CCAGCGCCACCTCACAGATGGAGGACCCAGTCTGTGGGAAAG**gg**GAGAC**tacag**CTTATCGGAACATATGGTTTCCTAAT

TCAAGTTAAAATGTCTGCACCCTGTTGCATCTGGTGCCATTTTAGTTAGTTAGAAATGCCACAC**gg**GGC**cacag**CTAAAG

GGGAAGAAACGGAGAAAAATACAGTTATT**gtgtg**CTCAAGAGCTGGGTTTTGCGGCTCATTAGCCAGTGGCCTC**tacag**C

T**ggcc**AGGTTTTG**ctgtc**GTTGCGGGGTCATTATGCCTTAATTTTTTTGCATAGTTATTCAGCCGTCACTTGAGGA
