# Supplementary material for: Dual transcriptional activities of PAX3 and PAX7 spatially encode spinal cell fates through distinct gene networks
Source: PLoS Biol. 2025 Oct 24;23(10):e3003448. doi: 10.1371/journal.pbio.3003448 (PMC12574859; doi:10.1371/journal.pbio.3003448)
Supplement: S4 Table — (DOCX) [file pbio.3003448.s011.docx]

Supplementary Table S4: Sequences of the guide RNAs (gRNA) used for generating the knock-out of Pax7^-/-^, Pax3^-/-^ and Pax3^-/-^; Pax7^-/-^ mESC lines, as well as the gRNA and donor DNA (ssDonor) for the generation of FLAG tag knock-in in Pax3 or Pax7 loci in mESC.

| **gRNA_Knock-Out** | **Sequence** |
| --- | --- |
| *pX459-Pax3sgExon1* | 5’-CGAGATCCGGAGAGTTCCCG-3’ |
| *pX459-Pax3sgExon3* | 5’-AGAACCCGGGCATGTTTAGC-3’ |
| *pX459-Pax7sgExon1* | 5’-GATTCGTCTCCAGCGTGCGC-3’ |
| *pX459-Pax7sgExon3* | 5’-AATCCGGGACCGGCTGCTGA-3’ |
| **gRNA_Knock-In** | **Sequence** |
| *pX459-Pax3sg385* | 5’-CGAGATCCGGAGAGTTCCCG-3’ |
| *pX459-Pax7sg616* | 5’-GTCTCCAGCGTGCGCAGGAA-3’ |
| **ssDonor_Knock-In** | **Sequence** |
| *ssDonor-Pax3* | 5’-  TTCGTCTCGCCTTCACCTGGATATAATTTGCGAGCGAAGCTGCCCCCAGGatggactataaggaccacgacggagactacaaggatcatgatattgattacaaagacgatgacgataagACCACGCTGGCCGGCGCTGTGCCCAGGATGATGCGGCCCGGCCCGGGGCA  -3’ |
| *ssDonor-Pax7* | 5’-CCAAGAGGTTTATCCAGCCGACTCTGGATTCGTCTCCAGCGTGCGCAGGAatggactataaggaccacgacggagactacaaggatcatgatattgattacaaagacgatgacgataagGCGGCGCTGCCCGGCGCGGTCCCCAGGATGATGAGACCCGGCCCGGGGCA-3’ |
