# Supplementary material for: Dual transcriptional activities of PAX3 and PAX7 spatially encode spinal cell fates through distinct gene networks
Source: PLoS Biol. 2025 Oct 24;23(10):e3003448. doi: 10.1371/journal.pbio.3003448 (PMC12574859; doi:10.1371/journal.pbio.3003448)
Supplement: S5 Table — Referenced chromosomal position of mouse Pax3: chr1: 78,197,134 and Pax7: chr4: 139,833,528. (DOCX) [file pbio.3003448.s012.docx]

**Supplementary Table S5:** Genotypes the three Pax7^-/-^, Pax3^-/-^ and Pax3^-/-^; Pax7^-/-^ mESC lines used in the study. Referenced chromosomal position of mouse Pax3: chr1: 78,197,134 and Pax7: chr4: 139,833,528.

| **Clone** | **Genotyping** | **Genotyping** |
| --- | --- | --- |
| A29 | *Pax3-/-* | Pax3∆288-3418/∆288-3418 |
| A37 | *Pax3-/-* | Pax3∆288-3418/∆291-418 |
| E8 | *Pax3 -/-* | Pax3 ∆304-3331/∆304-3331 |
| B16 | *Pax7-/-* | Pax7∆611-3994/∆605-3996 |
| B29 | *Pax7-/-* | Pax7∆611-3993/∆611-3993 |
| B34 | *Pax7-/-* | Pax7∆609-3998/∆609-3998 |
| F12 | *Pax3-/-; Pax7-/-* | Pax3∆304-3331/∆304-3331;  Pax7∆604-3994/∆604-3994 |
| I28 | *Pax3-/-; Pax7-/-* | Pax3∆283-3337/287-3342;  Pax7∆611-3993/∆604-3994 |
| I35 | *Pax3-/-; Pax7-/-* | Pax3∆282-3335/∆282-3335;  Pax7∆604-4004/∆604-4004 |
