# Supplementary material for: Dual transcriptional activities of PAX3 and PAX7 spatially encode spinal cell fates through distinct gene networks
Source: PLoS Biol. 2025 Oct 24;23(10):e3003448. doi: 10.1371/journal.pbio.3003448 (PMC12574859; doi:10.1371/journal.pbio.3003448)
Supplement: S6 Table — (DOCX) [file pbio.3003448.s013.docx]

## Supplementary Table S6: List of antibodies

| **Antigens** | **Species** | **Dilution** | **Provider (reference)** | **RRID** |
| --- | --- | --- | --- | --- |
| ATOH1 | Mouse | 1/800 | Abcam (ab27667) | [AB_870587](https://www.antibodyregistry.org/AB_870587) |
| FOXP2 | Goat | 1/200 | Santa Cruz (sc-21069) | AB_2107124 |
| FOXD3 | Guinea pig | 1/5000 | From T. Müller and K. Birchmeier (Muller et al., 2005) | none |
| FOXO1 | Rabbit | 1/1000 | Cell Signalling Technology (C29H4; #2880) | AB_2106495 |
| GFP | chicken | 1/1000 | Abcam (ab13970) | [AB_90755](https://scicrunch.org/resources/data/source/nif-0000-07730-1/search?q=AB_90755&l=AB_90755) |
| βgalactosidase | mouse | 1/500 | Promega (Z378) | AB_2313752 |
| bHLHE22 | Rabbit | 1/100 | Abcam (ab204791) | none |
| HuC/D | Mouse | 1/1000 | Thermo Fisher Scientific (16A11) | AB_221448 |
| ISLET1/2 | Mouse | 1/50 | DHSB clone 39.4 | [AB_2314683](http://antibodyregistry.org/AB_2314683) |
| LHX2 | Goat | 1/250 | Santa Cruz (sc-19344) | [AB_2135660](https://www.antibodyregistry.org/AB_2135660) |
| LBX1 | Guinea pig | 1/10000 | From T. Muller and K. Birchmeier | AB_2532144 |
| LMX1b | Guinea pig | 1/10000 | From T. Muller and K. Birchmeier | AB_2893158 |
| NKX6.1 | Mouse | 1/50 | DSHB (F55A10) | [AB_532378](https://www.antibodyregistry.org/AB_532378) |
| OLIG3 | Guinea pig | 1/10000 | From T. Muller and K. Birchmeier | AB_2315006 |
| p27 | Mouse | 1/500 | BD biosciences (610241) | [AB_397636](https://www.antibodyregistry.org/AB_397636) |
| PAX2 | Rabbit | 1/500 | Thermo Fisher Scientific (716000) | AB_2533990 |
| PAX6 | Rabbit | 1/250 | Biolegend (PRB-278P) | AB_291612 |
| PAX3 | Mouse | 1/50 | DHSB (PAX3 sup.) | [AB_528426](http://antibodyregistry.org/AB_528426) |
| PAX7 | Mouse | 1/100 | Santa Cruz (sc-81648) | [AB_2159836](https://www.antibodyregistry.org/AB_2159836) |
| POU4F1 | Mouse | 1/400 | Chemicon (A5945) | none |
| PSMAD1/5/9 | Rabbit | 1/250 | Cell Signalling Technology (13820S) | AB_2493181 |
| SOX1 | Goat | 1/200 | R&D systems (AF3369) | none |
| SOX9 | Goat | 1/100 | R&D systems (AF3075) | [AB_2194160](https://www.antibodyregistry.org/AB_2194160) |
| TFAP2α | Rabbit | 1/100 | Santa Cruz (sc8975) | AB_2240215 |
| Mouse IgG | Donkey | 1/500 | Thermo Fisher Scientific (A-21202) | AB_141607 |
| Mouse IgG | Donkey | 1/500 | Thermo Fisher Scientific (A10037) | AB_11180865 |
| Mouse IgG | Donkey | 1/500 | Thermo Fisher Scientific (A32787) | AB_2762830 |
| Goat IgG | Donkey | 1/500 | Thermo Fisher Scientific (A-11056) | AB_2534103 |
| Rabbit IgG | Donkey | 1/500 | Thermo Fisher Scientific (A10042) | AB_2534017 |
| Rabbit IgG | Donkey | 1/500 | Thermo Fisher Scientific (A31573) | AB_2536183 |
| Guinea pig IgG | Donkey | 1/500 | Jackson ImmunoResearch (706-546-148) | AB_2340473 |
|  | | | | |
