# Supplementary material for: Dual transcriptional activities of PAX3 and PAX7 spatially encode spinal cell fates through distinct gene networks
Source: PLoS Biol. 2025 Oct 24;23(10):e3003448. doi: 10.1371/journal.pbio.3003448 (PMC12574859; doi:10.1371/journal.pbio.3003448)
Supplement: S8 Table — (DOCX) [file pbio.3003448.s015.docx]

## Supplementary Table S8: Primers sequences used for qRT-PCR on cDNA prepared from mESC derived EB and organoids.

| Primer | Sequence | Primer | Sequence |
| --- | --- | --- | --- |
| Fw-Cdx2 | 5’-TAGTCGATACATCACCATCAGG-3’ | Rev-Cdx2 | 5’-TGATTTTCCTCTCCTTGGCTCT-3’ |
| Fw-Dbx1 | 5’-CAAGCCAGACCGGAAAAAGC-3’ | Rev-Dbx1 | 5’-TTTGTGGGAAGGGTCTGCTC-3’ |
| Fw-Fgf5 | 5’-TGTACTGCAGAGTGGGCATC-3’ | Rev-Fgf5 | 5’-ACAATCCCCTGAGACACAGC-3’ |
| Fw-Klf4 | 5’-ATGTGAGAGAGTTCCTCACGCC-3’ | Rev-Klf4 | 5’-GGAAGGGAGAAGACACTGCG-3’ |
| Fw-Lbx1 | 5’-TCGCCAGCAAGACCTTTA-3’ | Rev-Lbx1 | 5’-GATTTTCGCCGTTTCTTG-3’ |
| Fw-Nkx1.2 | 5’-ACTGCCTTCACTTACGAGCA-3’ | Rev-Nkx1.2 | 5’-AAATTTTGACCTGCGTCTCC-3’ |
| Fw-Olig3 | 5’-TGCCTCGTCCCCTCTGTCCG-3’ | Rev-Olig3 | 5’-GGAAGCCGCTGCCCAGTTGT-3’ |
| Fw-Pax3 | 5’-AGTTCTATCAGCCGCATCC-3’ | Rev-Pax3 | 5’-AATCAGGTTCAGAGTCAATATCG-3’ |
| Fw-Pax7 | 5’-CATGAACCCTGTCAGCAATG-3’ | Rev-Pax7 | 5’-CACTCGGGTTGCTAAGGATG-3’ |
| Fw-Pou4f1 | 5’-GATGAAATTCTCTGCCACTT-3’ | Rev-Pou4f1 | 5’-CAGCTTTTCAACAACTTCAC-3’ |
| Fw-Prdm12 | 5’-TGCCCCAGAGCATGTGGATA-3’ | Rev-Prdm12 | 5’-TCATCCAGCTTCGGTGGTCT-3’ |
| Fw-Sox1 | 5’-AGCGTGCCTTTGATTTCTCT-3’ | Rev-Sox1 | 5’-GGGATAAGACCTGGGTGAGA-3’ |
| Fw-Tubb3 | 5’-GCCCGACAACTTTATCTTT-3’ | Rev-Tubb3 | 5’-GCACGACATCTAGGACTGA-3’ |
| Fw-Pax6 | 5’-ACAACCTGCCTATGCAACCC-3’ | Rev-Pax6 | 5’-GCGGAGGGGTGTAGGTATCA-3’ |
| Fw-Pou5f1 | 5’-AGTTGGCGTGGAGACTTTGC-3’ | Rev-Pou5f1 | 5’-CAGGGCTTTCATGTCCTGG-3’ |
|  | | | |
