# Supplementary material for: Dual transcriptional activities of PAX3 and PAX7 spatially encode spinal cell fates through distinct gene networks
Source: PLoS Biol. 2025 Oct 24;23(10):e3003448. doi: 10.1371/journal.pbio.3003448 (PMC12574859; doi:10.1371/journal.pbio.3003448)
Supplement: Attachment — Submitted filename: Rondon_et_al_Pointbypoint_Answer_18082025.pdf [file pbio.3003448.s018.pdf]

We thank the reviewers for their thoughtful and constructive feedback on our manuscript, "*Dual PAX3/7 transcriptional activities spatially encode spinal cell fates through distinct gene networks*." In response, we have revised the manuscript to include new data, additional analyses, and clarifications throughout the text and supplementary material. All changes are highlighted in blue.
These revisions refine and reinforce, but do not alter, the core message of the study: PAX3 and PAX7 direct neuronal diversity in the spinal cord through modular transcriptional activities—repression and activation—gated by BMP signalling. Our data support a model in which BMP acts as a permissive signal that defines the spatial domain for PAX-driven transcriptional activation, rather than functioning as a direct cofactor. This framework advances our understanding of how transcription factors integrate with morphogen gradients to organize spatial patterning.

Key additions include:

1) New *in vivo* data from *Pax3*$^{+/PAX7\text{-}FOXO1}$ mice, confirming that PAX7, like PAX3, can drive dorsal progenitors towards the most dorsal fates of the spinal cord at the expense of the intermediate ones (new Figures 2A,B, S2B–C).
2) Spatiotemporal mapping of BMP signalling, PAX-dependent transcriptional activity, and OLIG3 expression in mouse and chick embryos (New Figures 1, S1), demonstrating their co-localization and supporting a model in which BMP signalling defines the spatial domain of PAX-mediated patterning.
3) Gain- and loss-of-function experiments showing BMP restricts PAX activation domains in chick neural tubes (Figure 1D,E, S1C,D).
4) Bioinformatic analyses reveal that PAX-bound CRMs show no enrichment for SMAD DNA-binding motifs. However, near key developmental genes of the dp1–dp3 domains, SMAD recruitment sites are found in proximity to distinct CRMs where PAX factors are also recruited, suggesting spatial proximity but arguing against direct cooperative binding.
5) Expanded discussion of PAX3/7 roles and BMP cross-talk.
6) Improved figures, statistics, and clarified methods.

Altogether, these revisions strengthen the conceptual clarity of the manuscript, providing a more robust mechanistic view of how transcription factors modularity and signalling gradients intersect to control spinal cell fate decisions.

Reviewer #1: The interplay between extrinsic signalling cues and transcription factor activity in determining cell fate during embryogenesis remains a central question in developmental biology. In this manuscript, Rondon et al. investigate how the transcription factors PAX3 and PAX7, in concert with BMP signalling, contribute to the formation of distinct neural progenitor domains along the dorsal-ventral axis of the neural tube. Employing a combination of mouse genetics, embryonic stem cell-derived organoids, and genomic analyses, the authors demonstrate a crucial synergistic role for PAX3 and PAX7 in ensuring the balanced production of dp1-dp6 interneuron progenitor domains. They further identify downstream transcriptional targets regulated by PAX3/7 and BMP signalling. In addition, the study maps distinct chromatin states associated with these targets and PAX3/7 genomic binding, revealing key enhancer and silencer elements that mediate PAX-driven promotion of dorsal (dp1-3) fates and repression of more ventral identities. Overall, this work represents a significant contribution, supported by well-designed experiments, robust data, and thoughtful interpretation. I have a few comments/questions for the authors:

Major comments:
- Point 1.1: The authors propose a compelling model in which BMP signalling in the dorsal neural tube is interpreted through the action of PAX3/7 transcription factors, which recruit enhancer and silencer elements to mediate transcriptional outcomes. While this framework is conceptually appealing, the

precise mechanisms by which BMP signals are integrated into this regulatory architecture remain insufficiently defined. Specifically, how BMP activity interacts with PAX transcription factors at the genomic level to specify dorsal cell fates is still an open question. Although the authors discuss potential mechanisms—such as a BMP-induced PAX-ZIC partnership—this aspect of the model would benefit from direct experimental validation. For instance, genome-wide mapping of pSMAD1/5/9 binding sites in BMP-treated spinal organoids of various genotypes (e.g., PAX3/7 single and double knockouts) could elucidate how BMP signalling interfaces with PAX factors, both individually and in combination. In support of this approach, the observed enrichment of GC-rich motifs in PAX3/7-bound cis-regulatory modules may indicate co-binding with BMP-activated SMAD proteins, consistent with previous findings (Martin-Malpartida et al., 2017; PMID: 29234012).

We thank the reviewer for this insightful and constructive comment. We fully agree that the precise mechanisms by which BMP signalling is integrated into the transcriptional program driven by PAX3/7 remain incompletely understood and are central to unravelling dorsal neural tube patterning. In response to this key point—and a related concern raised by Reviewer 2 (see Point 2.8)—we undertook several experimental and computational approaches to address the role and mechanistic interface of BMP and PAX3/7 activities. These new data and analyses are now integrated into the revised manuscript and new main (Figure 1B-G) and supplementary figures (Figure S1A-D, S5E-G).

1. Demonstration that the spatial domain of PAX3/7-dependent transcriptional activation corresponds to that of BMP Signalling

To examine the spatial relationship between BMP signalling and PAX3/7 activity, we now present a more comprehensive set of *in vivo* and *in vitro* analyses.

- Building on our initial comparison between ***P34::tk::LacZ*** reporter activity and the expression of *OLIG3*—a known BMP-responsive marker of dp1–dp3 domains (i.e. [1])—we now extend this analysis across developmental stages from E9.0 to E11.5 (Figure 1B,Ci; Supplementary Figure S1A,B). At all stages examined, the *P34* reporter domain largely overlaps with OLIG3 expression, with gradients that display similar slopes and dorsal restriction. Minor differences in ventral extent were seen across samples and developmental stages likely reflect differences in protein stability, nuclear dynamics, or reporter turnover.
- To more directly relate PAX activity to BMP signalling, we compared the distribution of ***P34::tk::LacZ*** activity with the pattern of ***phosphorylated SMAD1/5/9*** (pSMAD1/5/9) in the **mouse** neural tube (Figure 1B,Cii), and compared in the chick neural tube the activity of the ***P34*** reporter with that of a ***BRE::mlp::Turquoise*** BMP reporter [2] (Figure 1D, 1Ei). In both models, the BMP and PAX activity domains overlap extensively in the dorsal neural tube.
- These results are consistent with data in spinal organoids from the previous version of the manuscript showing that ***P34*** activity is detected only in organoids treated with BMP4 (Figure 3E), and with chromatin data showing that the accessibility and activation of PAX3/7-bound cis-regulatory modules (CRMs) is observed only in BMP-treated organoids (Figure 5E–G).

In conclusion, these findings support a model in which BMP signalling delimits the spatial domain in which PAX3/7 can act as transcriptional activators.

2. New evidence for causality: BMP signalling licenses PAX3/7 transcriptional activity

To move beyond correlative observations and address causality, we investigated whether BMP signalling is both necessary and sufficient to enable PAX3/7-dependent transcriptional activation. Building on previous data showing that BMP4 treatment robustly activates the *P34::tk::LacZ* reporter in spinal cord organoids—whereas reporter activity is minimal in the absence of BMP (Figure 3E)—we now provide two *in vivo* functional experiments in the chick neural tube (Figure 1D,E, S1C,D):

- **Gain for BMP signalling:** Electroporation of a constitutively active BMP receptor (**ALK3CA**, [3]) ectopically activates the P34 reporter, which contains only PAX binding motifs, in ventral regions where PAX3/7 is present but normally inactive (Figure 1D, 1Eii).

- **Inhibition of BMP signalling**: Overexpression of the inhibitory SMAD **SMAD6** abolishes P34 activity, despite maintained PAX3/7 expression (Figure 1D, Eiii), demonstrating that BMP signalling is required for PAX-dependent transcription.

In summary, these results demonstrate that BMP signalling is both necessary and sufficient to enable PAX3/7-mediated transcriptional activation in vivo.

3. Investigating genomic-level interactions between BMP effectors and PAX3/7
We fully agree with the reviewer that examining the potential for co-recruitment of SMAD and PAX proteins at genome levels is key for understanding the mechanisms underlying BMP mediated regulation of PAX activity.

To address this, we first performed **CUT&Tag experiments targeting phosphorylated SMAD1/5/9** in spinal cord organoids at day 3 of differentiation and at 1 h after BMP4 treatment or without treatment—a time point corresponding to the peak of BMP signalling in this model (see [4]). Despite two independent trials, signal-to-noise ratios were consistently poor, even at known SMAD target *loci* (Referee Figure 1). Outside of robust sites such as *Id1* (Referee Figure 1i), only **237 peaks** were confidently called across replicates (Referee Figure 1ii–iii). This is in stark contrast to the **~6955 peaks** reported in ChIP-seq experiments for SMAD1/5/9 in mouse neural stem cells treated with BMP4 [5], and likely reflects **technical limitations of the CUT&Tag method for SMAD proteins in our hands.**

Given these technical limitations, we turned to **computational approaches** to explore potential SMAD–PAX3/7 interactions at the genome levels (see new Figures S5E–G):

- We performed **motif enrichment analysis** across PAX3/7-bound CRMs, using a comprehensive set of SMAD1/5/9 binding motifs identified by EMSA or ChIP-seq [6–9], including those described in Martin-Malpartida et al., 2017 (PMID: 29234012). None of the SMAD motifs were significantly enriched in PAX3/7-bound regions (adjusted p > or = 1e–6), suggesting a lack of shared binding logic at the sequence level (Figure S5E). The observed enrichment of GC-rich motifs in PAX3/7-bound cis-regulatory modules mentioned by the referee do not correspond to the GC rich SMAD binding sites described by Martin-Malpartida. As a positive control, we analysed the same motifs in SMAD1/5/9 ChIP-seq peaks from neural stem cells (NSCs) isolated from E13.5 mouse brains [5] (Figure S5E). Four out of five motifs showed strong enrichment (adjusted p < 1e–10), confirming both the validity of the motifs and the sensitivity of our pipeline.
- Assuming partial conservation of SMAD recruitment between brain NSCs and spinal progenitors, we compared SMAD1/5/9 ChIP-seq peaks (from NSCs [5]) to our PAX3/7-bound CRMs. While target genes such as *Id1, Msx1, Atoh1,* and *Wnt1* are regulated by both PAX3/7 and BMP signalling, **SMADs and PAX3/7 bind distinct genomic regions near these loci** (Figure S5G). Heatmaps of SMAD1/5/9 ChIP-seq signal centred on PAX-bound CRMs further confirmed that SMAD occupancy does not co-localise with PAX binding sites (Figure S5F).

4. Alternative models: indirect coordination via ZIC factors or post-translational regulation
In the revised Discussion, we further consider alternative mechanisms of coordination between BMP and PAX activity:

- **Relay model**: BMP-induced factors such as ZIC proteins may act downstream of SMADs to modulate PAX3/7 activity. Notably, ZIC motifs are significantly enriched in PAX-bound CRMs, and ZIC proteins are known to respond to BMP signalling.
- **Post-translational modulation:** The fact that BMP signalling impacts activity at PAX reporters like P34—which lack canonical SMAD or ZIC binding sites—suggests additional regulatory layers (e.g., chromatin context or PAX modification and/or interactions with specific factors).

- Point 1.2: In my view, the data presented in Figure 2E do not fully support the authors' claim that "loss of PAX7 had minimal impact on cell composition" (pg. 10). Notably, LHX2 expression appears to

be significantly upregulated in PAX7-null organoids compared to BMP4-treated controls, while LBX1 expression is significantly downregulated in PAX7-null organoids specifically in the presence of BMP4. In both cases, the effects of PAX7 loss are opposite to those observed in PAX3-null organoids under the same BMP4 conditions. This raises the possibility of a secondary functional divergence between PAX3 and PAX7 in regulating dorsal neural subtypes. One potential confounding factor is the pooling of data from different clones; variability among individual clones could influence the observed expression patterns. To address this, I recommend that the authors present the data for each clone separately across genotypes. This would clarify whether the observed effects are consistent or driven by clone-specific behaviour. Alternatively, the findings may suggest a previously underappreciated cross-repressive interaction between PAX3 and PAX7, particularly in the specification of dl1 interneuron fate. While the broader model supports cooperative activity between these two transcription factors, the data also point to possible PAX3- and PAX7-specific roles that merit further investigation.

We thank the reviewer for raising this important point regarding the differential contributions of PAX3 and PAX7 in dorsal spinal cord development (see also point2.3).

1. Per-clone analysis added
In line with the reviewer's suggestion, we have now separated the data by clone across genotypes in the revised Figure 3G, rather than overlaying them as in the previous version. A two-way ANOVA analysis revealed minimal clone-specific effects, with the main variance attributable to genotype. These statistical results are now clearly presented in Figure 3F-G, the figure legends, and associated Table S7.

As noted, the same genotype-driven differences persist, including the observation under BMP4 conditions that **LHX2 expression is increased** in *Pax7*$^{-/-}$ organoids compared to controls (Figure 3F, 3Gi)., and **LBX1 expression further is decreased** (Figure 3F, 3Gii). The relevant section on page 15 now reads: "The cellular profile of *Pax7*$^{-/-}$ organoids treated with BMP4 closely resembled that of controls, with a predominance of LHX2^{+} dI1 INs and very few LBX1^{+} dI4–dI6 Ins (Figure 3F, Gi–ii). However, some differences were noted: the percentage of LHX2^{+} dI1 interneurons exceeded 30% in *Pax7*$^{-/-}$ organoids, compared to typically less than 30% in controls. Conversely, the proportion of LBX1^{+} dI4–dI6 cells remained very low, generally below the 7% seen in Control (Figure 3F, Gi,ii)."

2. Interpreting the subtle differences between PAX3 and PAX7
We acknowledge that the reviewer's observations may suggest a degree of functional divergence between PAX3 and PAX7. However, our data overall support the view that these two transcription factors act largely **convergently**, with overlapping genomic binding and partially redundant functions.

We first **address the specific case of dI1 interneuron specification**, where some differences were observed:

- *Pax7* deletion leads to a modest increase in LHX2^{+} dI1 interneurons, while *Pax3* deletion causes a reduction, which is further exacerbated in the double knockout—indicating that both factors promote this fate, with PAX3 playing a more prominent role in the BMP context (Figure 3F,G).
- Expression PAX3-FOXO1 significantly increases dI1 neuron production, whereas PAX7-FOXO1 overexpression does not have a consistent effect on this populations (new Figure 2A, 2Bi) (for the description of this new data see below point 1.3).
- Importantly, in mouse embryos, *Pax7* loss does not impair dI1 generation, indicating that PAX7 is not strictly required for this subtype (Figure 2A, Bi).

These findings argue against a model of cross-repression, and instead suggest minor differences in transcriptional potency, particularly under BMP-driven conditions.

Beyond the dI1 phenotype, **multiple lines of evidence support the broader view of functional convergence between PAX3 and PAX7**:

- **Additive phenotypes in double knockouts:** The *Pax3; Pax7* double knockout phenotype is consistently more severe than either single mutant. This is observed in: i) Cell type specification (mouse *in vivo*: Figure 2; organoids: Figures 3G-F and S4), ii) Transcriptomic profiles (Figure 4C-D), iii) Chromatin accessibility (Figure S6). We have revised the corresponding Results sections to clarify how each single mutant compares to the DKO and to highlight these additive effects.
- **Highly overlapping chromatin recruitment:** Genome-wide binding profiles of PAX3 and PAX7 are broadly similar (Figure 5A), suggesting shared occupancy at regulatory elements and consistent with common target gene networks.

Taken together, our data support the conclusion that PAX3 and PAX7 function in a largely redundant and cooperative manner, with PAX3 assuming a predominant role under BMP signalling conditions, particularly in specifying dorsal interneuron subtypes.

3. Revised discussion section

This comment, together with Point 1.3, led us to revise the Discussion to more explicitly address the relative contributions of PAX3 and PAX7 in dorsal neural specification—while acknowledging minor differences, we primarily argue in favour of functional convergence, based on the full set of molecular and phenotypic data. However, within the context of BMP signalling, our data suggest that PAX3 plays a more prominent role (see also our response to Point 1.3).

Minor comments:

- Point 1.3: in page 8, the authors state that "the balance between the activator and repressor activities of PAX3 and PAX7, regulated along the DV axis, is crucial for shaping the generation of dorsal interneuron subtypes". In my opinion this is the case only for PAX3 as transcriptional activator/repressor versions of PAX7 were not really tested and the statement should be modified accordingly.

We agree that, in the original manuscript, the effects of replacing one *Pax3* allele with activator (PAX3-FOXO1) or repressor (PAX3-EnR) variants were tested directly. However, previous studies suggest that these constructs act in a dominant manner over both PAX3 and PAX7. For example, phenotypes in *Pax3* $^{GFP/PAX3\text{-}ENR}$ embryos closely resemble those of *Pax3; Pax7* double knockouts and not those of single Pax3 mutant, in neural crest–derived craniofacial tissues where both factors act as transcriptional activators [10]. Similarly, dominant effects have been observed during muscle development in *Pax3* $^{+/PAX3\text{-}FOXO1}$ mice and *Pax3* $^{GFP/PAX3\text{-}FOXO1}$ mice [11] (see Referee Figure 2A).

To directly address the reviewer's point, we now include new data from a *Pax3* $^{+/PAX7\text{-}FOXO1}$ knock-in mouse, in which one *Pax3* allele was replaced with a PAX7-FOXO1 transcriptional activator (Figure 2; Figure S2B,C). PAX7-FOXO1 induces phenotypes similar to PAX3-FOXO1, including ectopic FOXD3$^+$ dI2 neurons and reduced LBX1$^+$ and LMX1B$^+$ populations, though the effects are slightly milder (Figure 2A; 2Bi–vi). This demonstrates that PAX7 transcriptional activation can be read by dorsal progenitors and biases them toward more dorsal (dI2-like) fates at the expense of dI4–dI6 identities.

Additionally, overexpression of PAX7-EnR in the chick neural tube represses OLIG3 expression, similar to PAX3-EnR, supporting repressor activity for both factors (Referee Figure 2A). While these chick gain-of-function data are less definitive than the mouse genetic models, they provide further functional support.

We have revised the Discussion accordingly to clarify the relative contributions of PAX3 and PAX7 during spinal cord development.

- Point 1.4: In page 9, the authors claim incorrectly that Pou5f1-negative cells are pluripotent, this should be corrected to Pou5f1+

The reviewer is correct. Pluripotent cells are *Pou5f1 (Oct4)*-positive, not negative as previously stated. To track the transition from pluripotency to a caudal epiblast state, we used *Klf4* as a pluripotency marker, *Pou5f1* as a marker of both pluripotent and epiblast states, and *Fgf5* as a caudal epiblast marker. *Pou5f1* and *Fgf5* were also mislabelled in previous Figure S2A; this has now been corrected in Figure S3A.

The text has been revised accordingly and now reads:

"Spinal organoids transitioned from pluripotency (Klf4⁺; Pou5f1⁺; Fgf5⁻) to an epiblast state (Klf4⁻; Pou5f1⁺; Fgf5⁺) within 48 h…"

- Point 1.5: In page 9, the authors mention that "DV identities were modulated by BMP4 exposure between days 3-4", however Fig 2 shows that BMP treatment took place between days 3-5. The authors should amend/clarify.

The schematic in previous Figure 2A was misleading. BMP4 was applied for 24 hours starting on day 3. We have corrected the figure (see revised Figure 3A) to reflect this and updated the legend to specify the treatment window.

- Point 1.6: In Figure 2C. OLIG3+/bgal-negative cells are also shown -how do the authors explain their presence?

The presence of OLIG3⁺/β-gal⁻ cells in the original figure was due to a channel assignment error: the fluorescent signals for OLIG3 and β-galactosidase were inadvertently swapped. This has now been corrected to match the stated color code (see revised Figure 3E).

Following this correction, we quantified the overlap and found that ~80–90% of OLIG3⁺ cells also express β-gal (Figure 3E). The small fraction of OLIG3⁺/β-gal⁻ cells likely reflects transient or sub-threshold PAX3/7 transcriptional activity, which is consistent with *in vivo* observations (Figure S1A). As shown in the new Figure S1A, *P34::tk::LacZ* and OLIG3 expression domains largely overlap in the dorsal neural tube, though minor differences are observed depending on developmental stage—likely due to dynamic regulation or differential protein stability.

As noted by the reviewer, β-gal⁺/OLIG3⁻ cells are present (in mean they represent 5% of all cells in the organoid). These likely correspond to neural crest cells, which are known to exhibit strong PAX3/7-dependent transcriptional activity.

To clarify, we have revised the main text (page 13) as follows:

"Analysis of *P34::tk::nLacZ* transgene activity in spinal organoids confirmed that PAX3/7 transcriptional activation occurs in this model system only in the presence of BMP4 (Figure 3E). As in vivo, activity was primarily restricted to OLIG3⁺ progenitors (Figure 3E), with approximately 80% of OLIG3⁺ cells showing β-galactosidase expression (Figure 3E). A small subset of β-gal^{high}/OLIG3⁻ cells (~5%), likely corresponding to neural crest cells, was also detected in the organoids (arrowhead in Figure 3Ci). Thus, in spinal organoids, PAX3/7 transcriptional activity is largely confined to dp1 and dp3 progenitors, consistent with its spatial restriction in developing embryos (Figure 1B,C)."

- Point 1.7: I find Figure S2D confusing: are PAX3+ cells present in PAX3-/- null organoids? What is the explanation for this?

The confusion arose from a mislabeled panel in the previous Figure S2Dii (now Figure S3D), which was incorrectly interpreted as showing *Pax3*$^{-/-}$ organoids. This panel was in fact intended to show PAX3 expression in the absence of PAX7. The labeling has now been corrected.

As shown, PAX3 expression remains unaffected in *Pax7*$^{-/-}$ organoids, indicating no compensatory upregulation. In contrast, no PAX3⁺ cells were detected in *Pax3*$^{-/-}$ organoids derived from any of our

three independently generated knockout clones (Figure S3C), as expected. The figure legend and main text were already accurate and required no changes.

Reviewer #2: Rondon et al present a very substantial original manuscript demonstrating activator (dorsal) and repressor (ventral) functions of PAX3/7 in spinal progenitor patterning using mouse models and organoids. They relate activator functions to BMP responsiveness. This is a very well written manuscript which reports experiments that are both creative and rigorous. A minor concerns should be addressed:

- Point 2.1: The statement "My study does not require an ethics statement" is perplexing given the use of mice. This seems adequately covered in the methods section.
Thank you for pointing this out. The use of animals in this study was indeed approved by the French Ministry of Research and the local ethics committee (CEB11.2025), under authorization number 01416.02, titled: *"Study of the gene regulatory network controlling dorsal spinal cord development."* This information has now been added to refine the Material and Methods section of our manuscript.

- Point 2.2: The Pax3 loss of function mice would be expected to develop spina bifida (and excencephaly). This open neural tube phenotype is not apparent in the histology shown, but presumably was seen in later stage embryos or at different anatomical levels? Was the anatomical region analysed predictably outside of the range of this lesion? Did loss of Pax7 modify this phenotype?
This observation is well taken. *Pax3* loss-of-function embryos consistently exhibit spina bifida at lumbar levels [12], while the brachial and thoracic regions remain closed and morphologically preserved (Referee Figure 2Bii, ii'). In *Pax3; Pax7* double mutants, neural tube closure defects are more severe and typically extend into the thoracic region; however, the brachial neural tube remains closed in the majority of embryos (e.g., [12]; Referee Figure 2B). This pattern was consistent across all embryos analysed in our study (see Figure 2A).
In *Pax3*$^{+/PAX3\text{-}FOXO1}$ embryos, neural tube closure is preserved, consistent with prior reports [3]. In contrast, *Pax3*$^{+/Pax3\text{-}EnR}$ and, more notably, *Pax3*$^{GFP/Pax3\text{-}EnR}$ embryos exhibit spina bifida, though in most cases the lesion does not extend to the brachial level [13] see also Figure 3D in [14]).
Notably, the phenotypes identified at brachial levels were consistently observed at thoracic and lumbar levels as well (not shown).
To clarify this in the manuscript, we have updated the text as follows:
Page 9: "We focused our analysis on the brachial region of the spinal cord, which remains morphologically preserved in *Pax3* and *Pax3;Pax7* mutant embryos. Neural tube closure defects primarily affect the lumbar region in Pax3 mutants and extend rostrally into the thoracic region in double mutants [11], complicating the assessment of interneuron positioning in those areas."
Page 11: "As with the loss-of-function mutants, all analyses were performed at brachial levels, where the neural tube remains closed [11,13,14]."

- Point 2.3: The superimposition of Pax3-GFP on the p34::tk:nLacZ reporter is very elegant. My enthusiasm for it is diminished by the 'overcooked' BGal image in which the background between nuclei is 0. Is reporter activity diminished in Pax3-GFP/GFP embryos or KO organoids?
We appreciate the reviewer's comment and agree that the initial β-galactosidase image in the original Figure 1B was overexposed. This was partly due to high reporter activity in non-neural tissues such as neural crest and dermomyotome, which required elevated exposure settings and resulted in saturation and artificially low background in the neural tube.

To address this, we have replaced the image with a more balanced version (revised Figure 1B), which better reflects the full dynamic range of reporter expression. We also now include a temporal

series and quantification of *p34* reporter activity during spinal cord development (Figure S1A). These data consistently show that p34 activity is more dorsally restricted than PAX3 or PAX7 expression.

To independently validate this observation, we performed X-gal staining across developmental stages (Referee Figure 3), which confirmed the dorsal restriction of reporter activity. As these findings aligned with our quantitative data, we opted not to include the X-gal results in the main figures.

Regarding the role of PAX3, we observed a complete loss of p34 reporter activity in *Pax3*$^{GFP/GFP}$ embryos (revised Figure 1F), indicating that PAX3 is required for reporter activation. Due to the presence of the *LacZ* gene in both the *Pax7* null allele and the *P34::tk::nLacZ* transgene, we could not directly assess reporter activity in *Pax7* mutant embryos. To circumvent this, we co-electroporated a PAX7-EnR expression vector with the *P34::tk::nLacZ* transgene into the chick neural tube and observed complete repression of reporter activity—mirroring the effect of PAX3-EnR (Figure 1G). Conversely, overexpression of the PAX7-FOXO1 activator induced strong ectopic reporter activation, including in ventral regions, similar to the effect of PAX3-FOXO1 overexpression (Figure 1G).

Together, these data show that p34 reporter activity is dynamically regulated by PAX3 and PAX7, with PAX3 playing a central role, likely due to its earlier and more dorsal expression [15–17]. The revised manuscript now includes updated figures and additional text to clarify these findings.

- Point 2.4: Many endpoints in the results are 'normalised' to something, e.g. "normalised to those of heterozygous littermates". The methods used to generate normalised values need to be explained (e.g. for the het normalisation, simple methods such as a ratio versus hets does not appear to have been used).
We agree with the reviewer on the need to clarify our normalization strategy.
Obtaining the full set of genotypes—particularly *Pax3; Pax7* double knockouts, which occur at a frequency of ~1/16 per litter—was technically and logistically challenging, requiring over four years of breeding and embryo collection.
Even within litters harvested at defined timepoints (e.g., E11.5), we observed considerable variability in developmental stage (ranging from ~E11.0 to E11.75), which introduced significant variation in neuronal subtype counts, including among controls. However, all phenotypes described in the manuscript were visually evident within individual litters, supporting the robustness of the observed genotype-specific effects.
To account for staging and inter-litter variability, we applied a batch-wise normalization strategy: within each experimental series, values were scaled such that the mean value for heterozygous embryos matched the expected count at E11.5. This approach minimized technical variation while preserving biologically meaningful differences between genotypes.
Details of this normalization procedure have been added to the Methods section of the revised manuscript.

- Point 2.5: Can the authors comment on - or provide data to illustrate - the overlap between pSMAS1/5/8 signal and the regions of the spinal cord in which PAX proteins appear to have gene activation functions? The pSMAD domain in published images of the mouse neural tube appears more dorsally restricted than dp1-3 (e.g. in Escuin et al Dis Models Mech 2023).
As suggested, we examined the spatial relationship between BMP signalling (pSMAD1/5/9 immunoreactivity in mouse and *BRE::mlp::Turquoise* reporter activity in chick) and PAX-dependent transcriptional activity, using the *P34::tk::LacZ* reporter (Figure 1B, 1Cii, 1D and 1Ei in the revised manuscript). We also compared *P34* activity with OLIG3 expression—a BMP-responsive dp1–dp3 marker—across stages E9.0 to E11.5 (Figure S1A,B).

Overall, we find substantial overlap between BMP signalling, PAX3/7-dependent transcriptional activity, and OLIG3 expression domains. Minor ventral shifts in boundaries—typically by a single cell row—vary with developmental stage, detection method, and marker dynamics, but do not undermine their overall co-localization.

These findings support a model in which BMP signalling defines the spatial domain where PAX3/7 act as transcriptional activators, despite small variations in precise boundary alignment. Further discussion is provided in our response to Point 1.1.

- Point 2.6: Figure 2E: why are the axes cropped between 60-70% when there are no data points above 70%?

No data points in the original Figure 2E exceeded 70%. The y-axis was cropped to retain 100% as a visual reference, but we agree this may have been misleading. In the revised Figure 3G, the y-axis scale has been adjusted to better reflect the actual data distribution and improve clarity.

- Point 2.7: Figure 2B: The description of BMP4 changing PAX3/7 'expression' seems misleading. The % of cells expressing PAX3 has clearly increased (statistical comparison on the graph would be worthwhile), but the intensity per cell ('expression') appears unchanged. Using different units of measure - cell versus organoid - in the same figure panel is also confusing.

As suggested, we have now included statistical comparisons to support the observed increase in the percentage of PAX3^{+} cells per organoid, as well as in PAX3 expression levels per cell in BMP4-treated organoids compared to untreated controls (Figure 3C, Dii). Conversely, we also observed a slight decrease in PAX7 expression levels per cell in BMP4-treated organoids compared to untreated ones (Figure 3Di). These changes in protein expression levels are consistent with the RT-qPCR quantifications of *Pax3* and *Pax7* mRNA levels shown in Figure S3B.

In addition, we have clarified the y-axis labelling in the relevant panels to explicitly indicate whether values represent measurements per organoid or per cell, thereby addressing the confusion caused by mixed units within the same figure.

- Point 2.8: "Notably, p0-p1… markers were more strongly enriched among PAX-repressed genes, whereas dp4-dp6 lineage markers were predominantly found among PAX-induced genes (Figure 3D)." Where is the p0-p1 data in this figure panel?

Thank you for pointing this out. In the original version of Figure 3D, the term *"lineage"* was intended to encompass both neural progenitors and their corresponding interneurons (INs), but we agree that this was not sufficiently clear. Additionally, the label on the left panel of Figure 3D was incorrect: it read "V0–V1 NP" but should have been "p0–p1." We have now corrected the terminology in both the figure and its legend to avoid this confusion. These updates are reflected in the revised Figure 4E and 4F.

- Point 2.9: Consider using more figure panel labels in all figures. E.g. Figure 4 has A-E, but could easily be A-G to help the reader understand the different comparisons.

We appreciate the reviewer's suggestion and have revised the figures accordingly. Panels have been further subdivided, and all images are now individually labelled. The corresponding figure legends and in-text references have been updated to reflect these changes. We believe these adjustments improve clarity and help guide the reader more effectively through the different comparisons.

Referee Figures and Legends

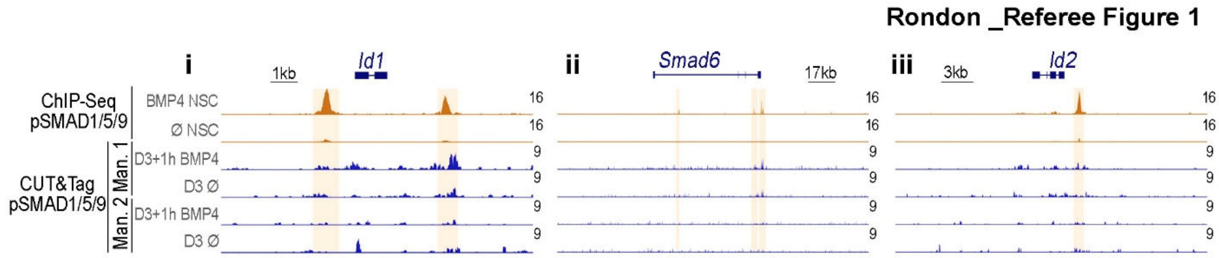

Figure 1: Comparison of CUT&Tag and ChIP-seq for pSMAD1/5/9 in spinal organoids. UCSC Genome Browser screenshots showing normalized ChIP-seq signals for pSMAD1/5/9 (dark orange tracks) from previously published datasets in brain-derived neural stem cells (NSCs) [18], alongside two normalized CUT&Tag tracks targeting pSMAD1/5/9 from two independent experiments (Man. 1 & 2, blue tracks) performed on spinal organoids (our data). A CUT&Tag peak is visible in one experiment (Man. 1) near the *Id1* gene (**i**), which overlaps with a ChIP-seq peak from the published dataset. However, most of the genome shows no detectable CUT&Tag peaks, even in regions near known pSMAD1/5/9 target genes such as *Smad6* and *Id2* (**ii**). Additionally, the CUT&Tag signal is generally noisy, with poor signal-to-noise ratio across the genome.

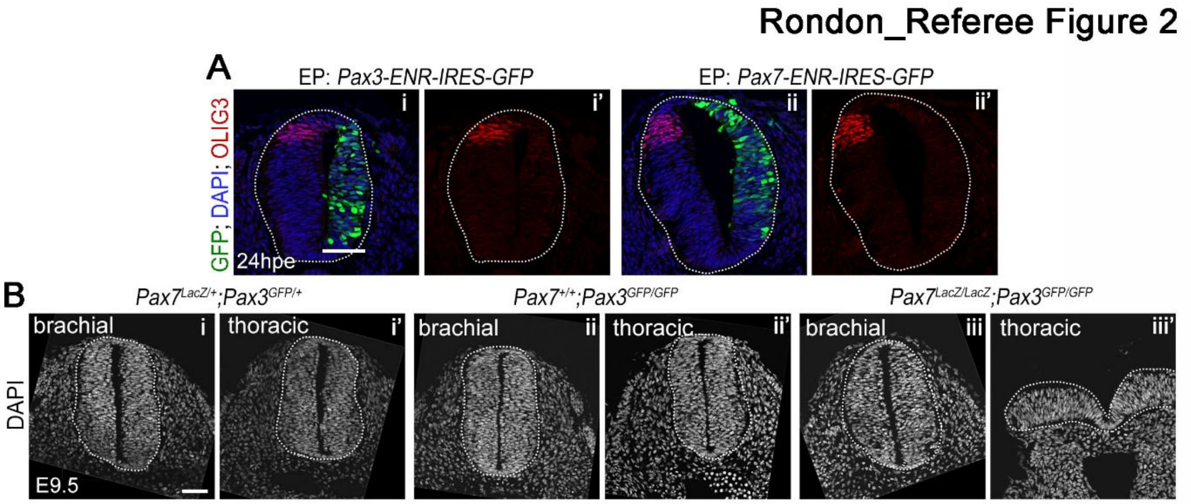

Figure 2: PAX3-ENR and PAX7-ENR inhibit OLIG3 expression and PAX3/PAX7 loss impairs neural tube closure at thoracic levels.

(A) Immunostaining for OLIG3 (red) and GFP (green) and DAPI-stained nuclei (blue) on transverse sections of chick embryos 24hours post-electroporation (hpe) with PAX3-ENR or PAX7-ENR expressing vectors. On the GFP^{+} electroporated side, OLIG3 expression is not observed, while it remains detectable on the non-electroporated GFP^{-} side. This indicates that the repressor variants PAX3-ENR (**i, i'**) and PAX7-ENR (**ii, ii'**) are both capable of blocking the emergence of dp1–dp3 progenitors. **(B)** DAPI-stained nuclei (blue) on transverse sections of E9.5 mouse embryos with the indicated genotypes at brachial and thoracic levels. At brachial levels, loss of Pax3 alone or combined loss of Pax3 and Pax7 does not impair neural tube closure (**ii, iii**). However, at thoracic levels, while Pax3 loss alone does not significantly affect closure (**ii'**), combined deletion of Pax3 and Pax7 leads to a failure in neural tube closure (**iii'**). In all images, white dotted lines outline neural tubes. Scale bars: 50 µm.

Rondon_Referee Figure 3

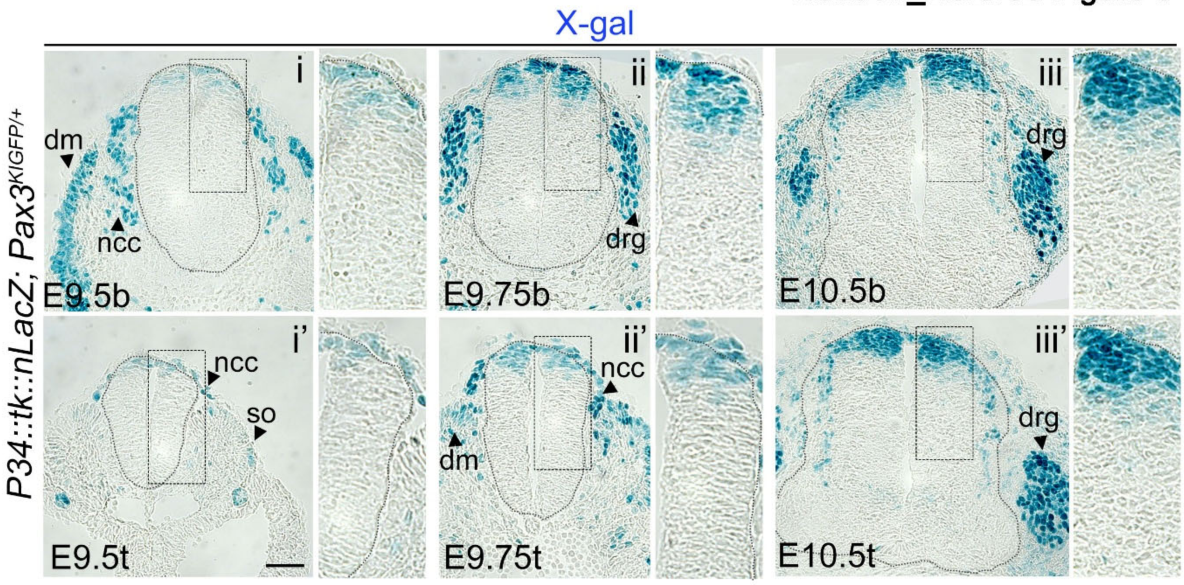

Referee Figure 3: Stable dorsal restriction of P34::tk::LacZ reporter activity during spinal cord development.

X-gal staining of β-galactosidase (β-gal) activity in transverse sections of brachial (b) and thoracic (t) spinal cords from *P34::tk::LacZ; Pax3$^{GFP/+}$* embryos at the indicated developmental stages. The β-gal signal, reflecting *P34* enhancer-driven expression, consistently localizes to the dorsal region of the developing spinal cord across stages. This dorsal restriction aligns with the expression patterns presented in the new Figure 1B, 1Cii–ii', and Figure S1A of our manuscript.

Methods not described in the manuscript: Histological slides prepared for immunostaining were incubated overnight at 37 °C in the following X-gal staining solution: 0.4 mg/mL X-gal (Sigma), 2 mM $MgCl_2$, 0.02% NP-40, 0.1 M PBS (pH 7.4), 20 mM $K_4Fe(CN)_6$, and 20 mM $K_3Fe(CN)_6$.

References Cited in the Response:

1. Zechner D, Müller T, Wende H, Walther I, Taketo MM, Crenshaw EB, et al. Bmp and Wnt/β-catenin signals control expression of the transcription factor Olig3 and the specification of spinal cord neurons. Developmental Biology. 2007;303: 181–190. doi:10.1016/j.ydbio.2006.10.045

2. Korchynskyi O, ten Dijke P. Identification and Functional Characterization of Distinct Critically Important Bone Morphogenetic Protein-specific Response Elements in the Id1 Promoter. Journal of Biological Chemistry. 2002;277: 4883–4891. doi:10.1074/jbc.M111023200

3. Timmer JR, Wang C, Niswander L. BMP signaling patterns the dorsal and intermediate neural tube via regulation of homeobox and helix-loop-helix transcription factors. Development. 2002;129: 2459–2472. doi:10.1242/dev.129.10.2459

4. Duval N, Vaslin C, Barata T, Frarma Y, Contremoulins V, Baudin X, et al. BMP4 patterns Smad activity and generates stereotyped cell fate organisation in spinal organoids. Development. 2019; dev.175430. doi:10.1242/dev.175430

5. He L, Wen J, Dai Q. PRDM16 functions as a co-repressor in the BMP pathway to suppress neural stem cell proliferation. eLife Sciences Publications, Ltd; 2025. doi:10.7554/elife.104076.2

6. Kim J, Johnson K, Chen HJ, Carroll S, Laughon A. Drosophila Mad binds to DNA and directly mediates activation of vestigial by Decapentaplegic. Nature. 1997;388: 304–308. doi:10.1038/40906

7. Morikawa M, Koinuma D, Tsutsumi S, Vasilaki E, Kanki Y, Heldin C-H, et al. ChIP-seq reveals cell type-specific binding patterns of BMP-specific Smads and a novel binding motif. Nucleic Acids Res. 2011;39: 8712–8727. doi:10.1093/nar/gkr572

8. Kusanagi K, Inoue H, Ishidou Y, Mishima HK, Kawabata M, Miyazono K. Characterization of a Bone Morphogenetic Protein-responsive Smad-binding Element. Heldin C-H, editor. MBoC. 2000;11: 555–565. doi:10.1091/mbc.11.2.555

9. Martin-Malpartida P, Batet M, Kaczmarska Z, Freier R, Gomes T, Aragón E, et al. Structural basis for genome wide recognition of 5-bp GC motifs by SMAD transcription factors. Nat Commun. 2017;8: 2070. doi:10.1038/s41467-017-02054-6

10. Zalc A, Rattenbach R, Auradé F, Cadot B, Relaix F. Pax3 and Pax7 Play Essential Safeguard Functions against Environmental Stress-Induced Birth Defects. Developmental Cell. 2015;33: 56–66. doi:10.1016/j.devcel.2015.02.006

11. Relaix F, Polimeni M, Rocancourt D, Ponzetto C, Schäfer BW, Buckingham M. The transcriptional activator PAX3-FKHR rescues the defects of *Pax3* mutant mice but induces a myogenic gain-of-function phenotype with ligand-independent activation of Met signaling in vivo. Genes Dev. 2003;17: 2950–2965. doi:10.1101/gad.281203

12. Sudiwala S, Palmer A, Massa V, Burns AJ, Dunlevy LPE, De Castro SCP, et al. Cellular mechanisms underlying Pax3 related neural tube defects and their prevention by folic acid. Disease Models & Mechanisms. 2019; dmm.042234. doi:10.1242/dmm.042234

13. Bajard L, Relaix F, Lagha M, Rocancourt D, Daubas P, Buckingham ME. A novel genetic hierarchy functions during hypaxial myogenesis: Pax3 directly activates *Myf5* in muscle progenitor cells in the limb. Genes Dev. 2006;20: 2450–2464. doi:10.1101/gad.382806

14. Lagha M, Sato T, Regnault B, Cumano A, Zuniga A, Licht J, et al. Transcriptome analyses based on genetic screens for Pax3 myogenic targets in the mouse embryo. BMC Genomics. 2010;11: 696. doi:10.1186/1471-2164-11-696

15. Goulding MD, Chalepakis G, Deutsch U, Erselius JR, Gruss P. Pax-3, a novel murine DNA binding protein expressed during early neurogenesis. The EMBO Journal. 1991;10: 1135–1147. doi:10.1002/j.1460-2075.1991.tb08054.x

16. Moore S, Ribes V, Terriente J, Wilkinson D, Relaix F, Briscoe J. Distinct Regulatory Mechanisms Act to Establish and Maintain Pax3 Expression in the Developing Neural Tube. Desplan C, editor. PLoS Genet. 2013;9: e1003811. doi:10.1371/journal.pgen.1003811

17. Kicheva A, Bollenbach T, Ribeiro A, Valle HP, Lovell-Badge R, Episkopou V, et al. Coordination of progenitor specification and growth in mouse and chick spinal cord. Science. 2014;345: 1254927. doi:10.1126/science.1254927

18. He J, Huo X, Pei G, Jia Z, Yan Y, Yu J, et al. Dual-role transcription factors stabilize intermediate expression levels. Cell. 2024;187: 2746-2766.e25. doi:10.1016/j.cell.2024.03.023
